# A Systematic Review Examining the Experimental Methodology Behind In Vivo Testing of Hiatus Hernia and Diaphragmatic Hernia Mesh

**DOI:** 10.1007/s11605-021-05227-3

**Published:** 2021-12-21

**Authors:** Thomas Whitehead-Clarke, Victoria Beynon, Jessica Banks, Rustam Karanjia, Vivek Mudera, Alastair Windsor, Alvena Kureshi

**Affiliations:** 1 Centre for 3D Models of Health and Disease, Division of Surgery and Interventional Science, University College London, Charles Bell House, Foley Street, London, W1W 7TY UK; 2 Ashford and St Peter’s NHS Trust, Stanwell, UK; 3 Arrowe Park Hospital, Birkenhead, UK; 4 Princess Grace Hospital, London, UK

**Keywords:** Hiatus, Hernia, Mesh, In vivo, Testing

## Abstract

**Introduction:**

Mesh implants are regularly used to help repair both hiatus hernias (HH) and diaphragmatic hernias (DH). In vivo studies are used to test not only mesh safety, but increasingly comparative efficacy. Our work examines the field of in vivo mesh testing for HH and DH models to establish current practices and standards.

**Method:**

This systematic review was registered with PROSPERO. Medline and Embase databases were searched for relevant in vivo studies. Forty-four articles were identified and underwent abstract review, where 22 were excluded. Four further studies were excluded after full-text review—leaving 18 to undergo data extraction.

**Results:**

Of 18 studies identified, 9 used an in vivo HH model and 9 a DH model. Five studies undertook mechanical testing on tissue samples—all uniaxial in nature. Testing strip widths ranged from 1–20 mm (median 3 mm). Testing speeds varied from 1.5–60 mm/minute. Upon histology, the most commonly assessed structural and cellular factors were neovascularisation and macrophages respectively (*n* = 9 each). Structural analysis was mostly qualitative, where cellular analysis was equally likely to be quantitative. Eleven studies assessed adhesion formation, of which 8 used one of four scoring systems. Eight studies measured mesh shrinkage.

**Discussion:**

In vivo studies assessing mesh for HH and DH repair are uncommon. Within this relatively young field, we encourage surgical and materials testing institutions to discuss its standardisation.

**Supplementary Information:**

The online version contains supplementary material available at 10.1007/s11605-021-05227-3.

## Introduction

A hiatus hernia (HH) is defined as the protrusion of an organ (typically the stomach) through the oesophageal hiatus. They are generally sub-categorised into types I–IV depending on the position of the gastro-oesophageal junction as well as the gastric fundus and other viscera.^1^ Together with the presence or absence of symptoms, the type of HH will be a key factor behind the decision for surgical treatment. Type I “sliding” hernias will generally only be repaired if symptomatic.^1,2^ Types II–IV or so called “paraoesophageal” hernias are less common, but carry a higher risk of future complications.^1^ As such, these are more likely to be surgically managed even if asymptomatic. Diaphragmatic hernias (DH) are a different pathology involving the protrusion of viscera through a primary defect in the diaphragm. This pathology can either occur congenitally or secondary to trauma, and urgent surgical repair is indicated in both.^3,4^

Surgical repair of HH has been practised for many years, and typically involves reduction of the hernia, excision of the hernia sac, approximation of the crus and a concomitant fundoplication procedure. Some surgeons will choose to augment this cruroplasty with the placement of a mesh—usually encircling the oesophagus. Congenital DH should be repaired with a mesh patch if primary approximation is not possible.^3^

Similar to conventional hernia mesh, clinicians and medical device companies continue to search for the ideal material for the purpose of repairing DH and HH. Whilst mesh use for conventional hernia repair is not controversial, mesh repair for HH is made contentious by the dynamic nature of the diaphragm and its relationship with the oesophagus. Some reviews have claimed that mesh use may be helpful in the prevention of recurrence when treating large hiatus hernias,^5^ whereas a more recent systematic review has found no significant advantage in the use of mesh.^6^

Whether or not mesh augmentation is helpful, its use is certainly associated with complications such as migration and erosion.^7,8^ Reviews of the literature have highlighted a variety of ways to manage such complications, some resulting in gastrectomy or oesophagectomy. Whilst such complications are not an issue for congenital DH repair, other problems such as recurrence and visceral adhesions may have serious consequences given the young patient cohort.

Given these potential complications behind mesh materials, pre-clinical in vivo testing represents an essential tool in optimising performance and is a legal requirement across the world. Following our group’s recently published review of hernia mesh testing, which revealed a significant variation in studies,^9^ we have conducted a new systematic review of in vivo studies assessing meshes for HH and DH repair.

## Methods

### Registration

This systematic review was registered through the international prospective register of systematic reviews (PROSPERO) and given ID number CRD42021231744. A protocol was developed and submitted to PROSPOERO as per the PRISMA guidelines.

### Literature Search

The OVID interface was used to conduct a search of Medline and Embase databases. The purpose of the search was to identify in vivo studies in which mesh prostheses were implanted into animal subjects for the purpose of repairing HH or DH. The prosthesis and/or surrounding tissue should be subsequently extracted for the purposes of testing. Articles in the English language were selected between January 2000 and December 2021. Specific search terms used for both Embase and Medline can be found in our supplementary material (supplementary Fig. 1).

### Article Screening

Our initial search produced 62 articles; of which, 18 duplicates were excluded. Details of all remaining 44 papers were uploaded to Covidence online systematic review software (Covidence systematic review software, Veritas Health Innovation, Melbourne, Australia, www.covidence.org). Using this platform, the remaining articles underwent abstract review. Abstracts were assessed by 4 of the authors (T.W.C., V.B., J.B., R.K.). Each abstract was screened independently by two reviewers and was automatically included or excluded if there was consensus. In the case of disagreement, the final decision was referred to the lead author (T.W.C.). Specific inclusion/exclusion criteria were disseminated amongst the authors to standardise the process. Following abstract review, 22 articles were excluded. The remaining 22 papers were distributed equally between the four authors (T.W.C., R.K., J.B., V.B.) for full-text review and data extraction. During this final stage, 4 further papers were excluded. Full details of the inclusion and exclusion criteria and selection process are provided below.

#### Inclusion Criteria

- In vivo studies
- Single arm studies and comparative studies that look to assess the performance of mesh used for the repair of hiatus or diaphragmatic hernias
- Studies where the mesh and tissue sample is explanted from the animal for testing. The only exception being adhesions which can be tested in vivo
- Studies looking at any type of mesh or novel coating including both synthetic and biological meshes
- Studies that examine a mesh/tissue sample for inflammatory, structural, adhesional or biomechanical properties, as well as those that assess mesh shrinkage
- Studies published between January 2000 and December 2020 inclusive
- Studies published in the English language

#### Exclusion Criteria (in Order of Relevance)

- Human studies
- In vitro studies
- Ex vivo studies (where meshes are not implanted into a living animal)
- Studies where the primary subject of investigation is not mesh performance
- Studies that compare or assess different fixation techniques or adjuncts that are not meshes
- Studies testing new pharmacological products
- Studies that only measure systemic inflammation in the form of inflammatory markers
- Studies that only assess the extent to which a mesh is resistant/susceptible to infection

**Fig. 1:**
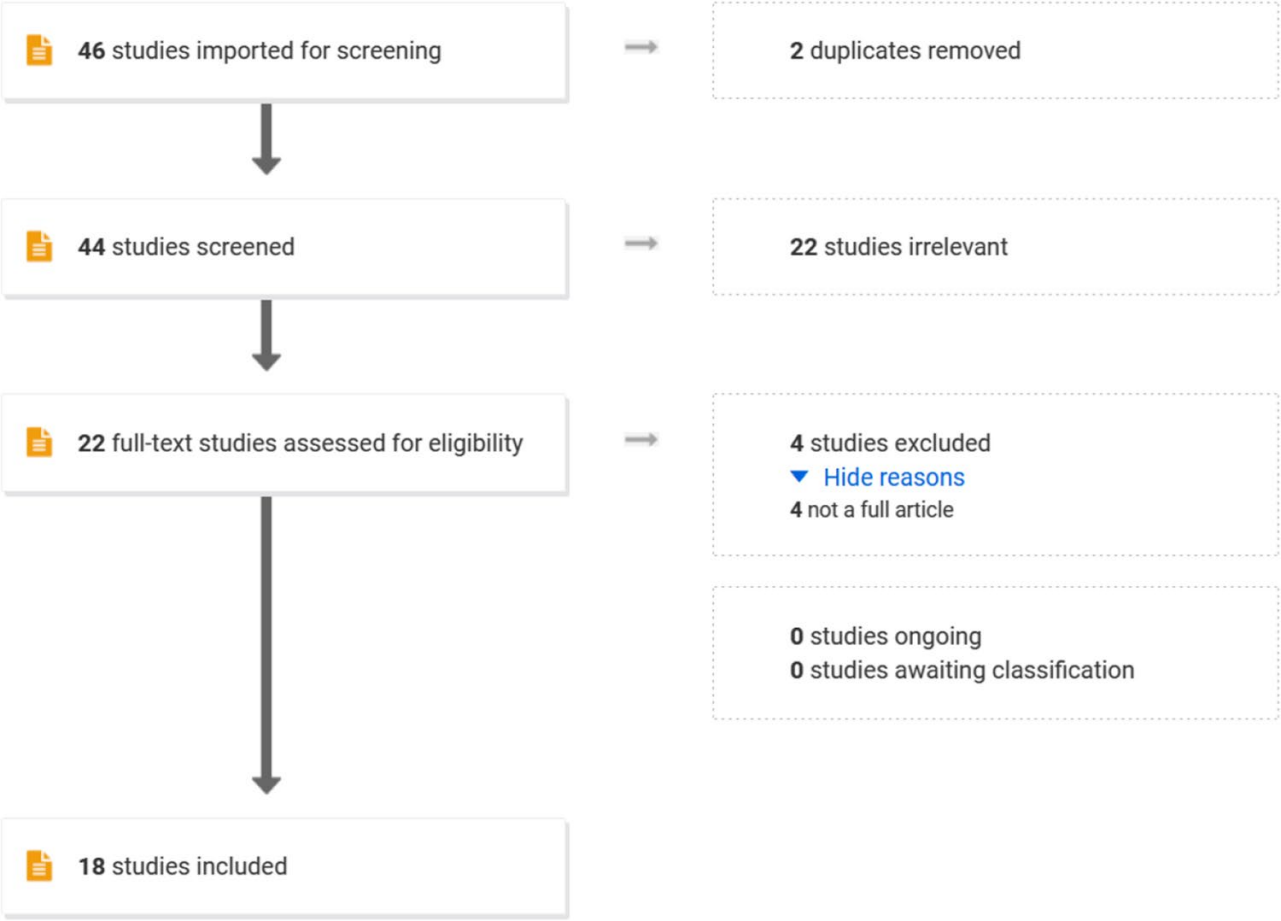
PRISMA diagram of abstract and full-text review

A summary of the abstract and full-text screening process can be seen in Fig. 1.

### Data Extraction

Data extraction was undertaken using a standardised online spreadsheet. Before data extraction began, a meeting was held between the four authors responsible for data extraction (T.W.C., V.B., J.B., R.K.) to standardise the process. The same four authors from our group had previously collaborated on a similar project,^9^ which created a good understanding of the process. Data collection forms were adapted during the process to reflect the results being collected. As new relevant variables came to light, the standardised form was adjusted, and alterations made to our standardised data collection form and our online protocol. A full list of the categories and variables that were measured is outlined below.

#### Study/Experimental Data

- Pathology mimic (hiatus/ diaphragmatic hernia)
- Study with/without non-mesh control
- Animal species used
- Animal subspecies used
- Number of animals used
- Weight of animals used (mean/range)
- Age of animals (mean/range)
- Defect formed in diaphragm (yes/no)
- Shape of defect (linear/2D)
- Nature of defect (acute/chronic)
- Size of mesh overlap
- Defect closure (yes/no)
- Time between mesh implantation and explantation

#### Mechanical Testing

- Was this assessed?
- Type of testing (Uniaxial/ball burst, etc.)
- Testing speed (mm/minute)
- Units of measurement taken (pascals/Newtons, etc.)
- Width of testing strips
- Thickness of testing strips
- Constitution of testing strips (with regards to mesh and/or tissue tested)
- Inter-clamp distance

#### Histology—Structural Assessment

- Was the interaction between oesophagus and mesh assessed?
- Was collagen assessed
- How was collagen measured
- Other structural factors assessed (vascularisation/fibrosis/inflammation/new tissue formation, etc.)
- How were factors visualised (staining/microscopy)
- How were factors measured (qualitatively/quantitatively with or without a scoring system)

#### Histology—Inflammatory Cellular Analysis

- Was the interaction between oesophagus and mesh assessed?
- Which cells were assessed (macrophages/leucocytes/fibroblasts, etc.)

How were factors visualised (staining/microscopy)

How were factors measured (qualitatively/quantitatively with or without scoring system)

#### Assessment of Adhesions

- Was the interaction between oesophagus and mesh assessed?
- Were adhesions assessed?
- How were they assessed? (scoring system/mechanical testing, etc.)

#### Assessment of Mesh Shrinkage

- Was this assessed?
- How was this assessed? (scoring system/reduction in size, etc.)

## Results

In total, 18 studies were reviewed. Results are divided into six sections as described in our methods.

### Experimental Methods

Of the 18 studies, 9 used an in vivo model of HH repair, whilst 9 others used a model of DH repair. The most common animal models used were pigs (*n* = 7) and rabbits (*n* = 7) then dogs (*n* = 2) and finally sheep (*n* = 1) and rats (*n* = 1). Animal size was not reported in 2 of the studies and 11 did not report the animals’ age. One study failed to report either. A non-mesh control was only used in 6/18 studies.

Of the 9 DH mesh studies, 8/9 had a defect made in the diaphragm. One study closed the defect with sutures before mesh application, and another used closure for only some animals. Median defect size was 1.5 cm^2^, and mean mesh overlap was 1 cm. All repairs were undertaken during the same operation as defect formation. Explantation of mesh and tissue took place from 30–180 days. Four studies explanted tissue at multiple time points; 5 studies at one single time point.

Of the 9 hiatal studies, 3/9 deliberately produced a more prominent hiatal defect (around 4 cm width) and 6/9 involved cruroplasty. All hernia models were produced and repaired in a single procedure, apart from one study in which the hernia model was allowed to mature over time before repair in a later procedure. Explantation of tissue took place anywhere from 4 weeks to 1 year, but all studies had a single time point for tissue explantation.

### Mechanical Testing

**Fig. 2:**
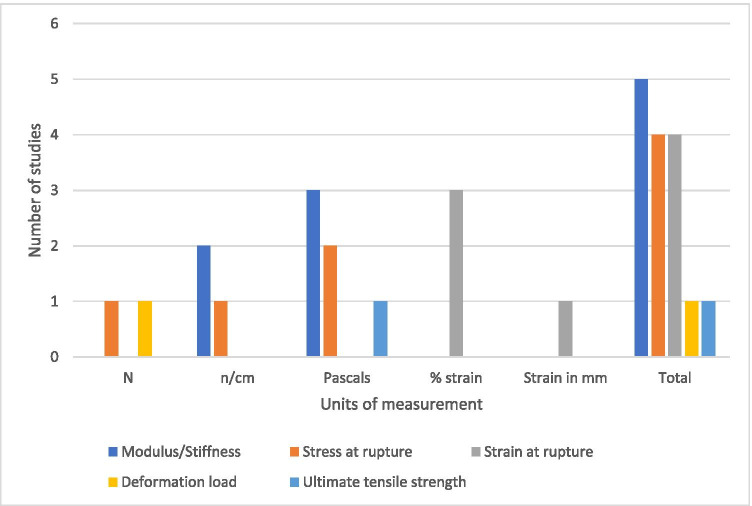
Number of studies in which different mechanical testing variables were measured and their units

Mechanical testing of tissue samples was undertaken in 5 of the 18 studies. All mechanical testing took the form of uniaxial tensile strength testing. The most common factor measured was the stiffness or modulus of the tissue (*n* = 5). This was followed closely by the strain at rupture (*n* = 4) and stress at rupture (*n* = 4). Full details of the mechanical testing variables can be seen in Fig. 2.

The width of testing strips was documented in every study (median 3 mm, 1–20 mm). Depth or thickness of testing strips was recorded in one of the studies, and speed of testing recorded in 3 of the studies. Only one of the studies described the nature of the tissue that was being tested mechanically. Full details of these mechanical testing data can be seen in Table 1.

**Table 1:** A summary of mechanical testing variables during uniaxial testing in 5 studies

Authors	Variables			
	Thickness of strip (mm)	Width of strip (mm)	Inter-clamp distance (mm)	Testing speed (mm/minute)
Altieri et al.^10^	1–2 (varying)	1	Unknown	Unknown
Böhm et al.^11^	Unknown	20	20	10
Mayer et al.^12^	Unknown	10	15	60
Amigo et al.^13^	Unknown	3	Unknown	1.5
Eastwood et al.^14^	Unknown	2	Unknown	Unknown

### Histology—Structural Assessment

**Fig. 3:**
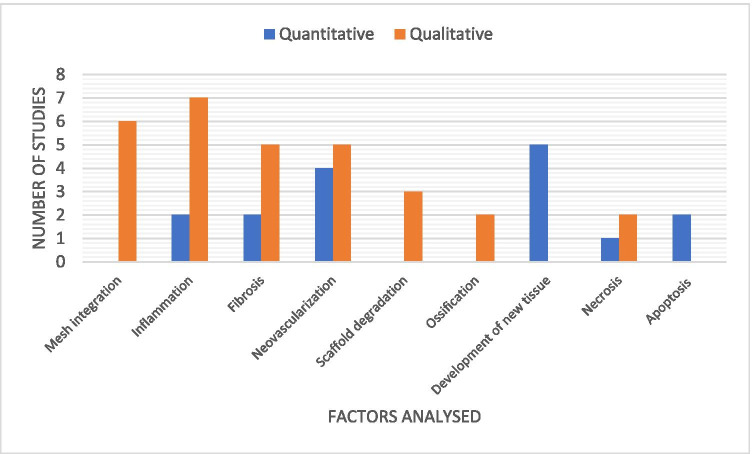
The number of studies analysing various structural factors on histology

The vast majority of studies (17/18) carried out a histological structural assessment of the tissue. The presence or absence of collagen was reported in 10 studies, of which 5 assessed collagen abundance and 5 described collagen organisation or alignment (1 study assessed both). The ratio of type I and type III collagen within the sample was examined by 5 of the studies. The most common histological assessments were that of that of inflammation and neovascularisation (*n* = 9). Qualitative analysis was twice as common as quantitative analysis. Scoring systems of various types were used in 10 studies. Of those, 2 studies described the use of a previously published scoring system. With regard to HH studies, 7/9 assessed interaction between implants and the oesophagus. Further results are provided in Fig. 3.

### Histology—Inflammatory Cellular Analysis

**Fig. 4:**
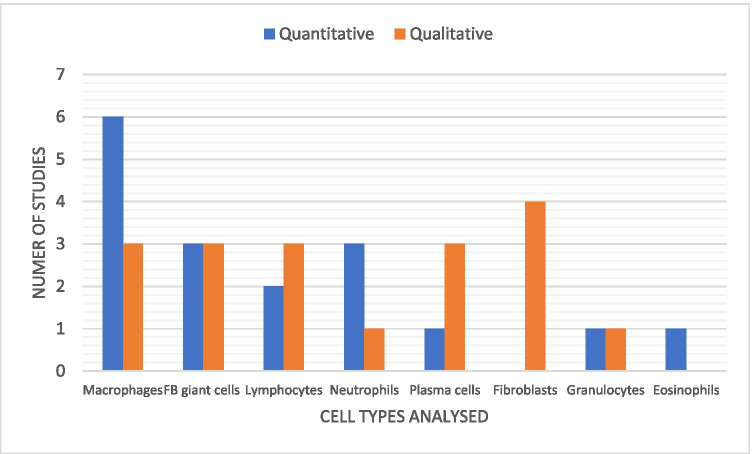
The number of studies analysing various cellular factors on histology

Of the HH papers reviewed, 6/9 papers assessed the cellular inflammatory reaction between the mesh and the oesophagus. Out of all 18 papers, 12 analysed the presence or abundance of inflammatory cells around mesh implants. The most commonly assessed cells were macrophages (*n* = 9) followed by foreign body giant cells^6^ and lymphocytes.^5^ There was an equal division between cells assessed quantitatively and qualitatively (6 each). Scoring systems were used to assess inflammatory cellular activity in 8 articles, only one of which described a system previously used in the literature. Further results are provided in Fig. 4.

### Assessment of Adhesions

The development of adhesions was assessed in 11 articles, of which 3 studies provided a qualitative description of adhesions and 8 others used a scoring system. Four different scoring systems were cited from the literature.^15–18^

### Assessment of Mesh Shrinkage

Mesh shrinkage was assessed in 8 studies, 6 of which reported the relative reduction in mesh size. One further study discussed their findings qualitatively and another summarised their findings using their own binary scoring system.

## Discussion

Over the 20 years reviewed, 18 in vivo DH or HH mesh testing studies were conducted. Unlike previous studies that reviewed in vivo models of ventral hernia repair,^9,19^ porcine models were used most frequently (39% of studies) and rat models were least frequent (6% of studies). This finding is reassuring given that porcine models should provide a more accurate representation of human physiology.

The majority of studies (94%) provided sizes of the animals used; however, the majority also failed to document the animals’ age (61%). Whilst potentially trivial, such details are important in the testing of certain biomaterials, particularly those used in congenital DH. DH patients are frequently neonates and therefore rapidly growing. Certain studies keep this at their forefront, making reference to a “growing animal model” and noting animals’ significant increase in size over the course of the study.^20^ Given that materials for paediatric use will need to work through periods of rapid growth, the use of younger (and therefore growing) animal models may provide more representative results.

Unlike previous work,^9^ the comparators used for uniaxial tensile testing were mostly consistent. One hundred percent of mechanical testing studies measured tissue modulus and 80% measured the stress or strain at point of rupture. Concerningly, however, there remains significant variation in the units used to measure these factors. Stress at rupture, for example was measured with 3 separate units (N, N/cm and Pascals). Whilst these units are translatable between one another, such translation depends upon knowing both the testing strip width and thickness. Given that none of the studies clearly documented testing strip thickness, it is impossible to compare the results of different studies.

Mechanical testing speed also varied greatly, ranging between 1.5 and 60 mm/minute. This sort of variability in testing speeds makes it difficult to establish a large-scale data set for the purposes of systematic review. Whilst no guidelines exist for the testing of such samples, there are guidelines available for the tensile testing of soft plastics from ASTM international—suggesting testing speeds of either 50 mm/500 mm per minute.^21^ Most importantly perhaps, only 1 study gave an exact description of what their testing strips were made of (mesh/tissue interface, tissue/tissue interface, etc.). Such a lack of clarity leaves it difficult to assess what is actually being tested—whether that be the in-growth of the mesh or the healing between the tissues.

In vivo testing of mesh devices is a vital step in preventing serious complications and with HH mesh, no complication is more serious than visceral erosion. Several recent literature reviews describe a variety of cases of hiatal mesh erosion and migration^7,8^ and that such erosion is more common with the use of synthetic meshes.^7^ It is perhaps reassuring then that the majority of HH studies in our work analysed the histological reaction between mesh and oesophagus. Surgeons continue to explore ways of avoiding such complications including the use of a bioresorbable mesh^22^ or novel techniques such as those described by Braghetto et al.^23^. Using this technique, the hernia sac is dissected out, brought into the abdominal cavity and wrapped around the abdominal oesophagus to prevent mesh erosion.

The ultimate prevention is of course to avoid mesh altogether, and its role in HH repair is still not a matter of consensus. The most recent systematic review on the subject from Petric et al. reviewed 7 randomised controlled trials. They concluded that there was no clear advantage to the use of hiatus hernia mesh in the reduction of recurrence compared to a simple suture repair.^6^ The two techniques were also found to be similar in terms of patient satisfaction and functional outcomes, with the only substantial difference being operative time. Another systematic review from 2013 specifically identified that the use of biological mesh provided no reduction in recurrence rates when compared to suture repair.^24^ Two separate systematic reviews have, however, suggested that if the hiatus hernia is particularly large, then mesh augmentation may help reduce recurrence rates.^5,25^

DH meshes have a different complication profile, with issues such as recurrence and adhesion formation being of higher relevance. Given the young age of congenital DH patients, such complications can still incur serious consequences. Certain current literature suggests that patch repair with a mesh should only be undertaken for congenital DH if direct closure has been unsuccessful.^3^ However, if a mesh is required, ideal product selection remains an issue of some debate. In an international survey of paediatric surgeons in 2016, Gore-tex was the most frequently used material for congenital DH repair,^26^ rather than other biological materials. This pattern appears to be validated by a recent study suggesting that porcine dermal patches are a significant factor for recurrence when compared to Gore-tex patches.^27^ There is however no complete consensus; a meta-analysis from 2012 concluded that there is no difference in recurrence rates between the use of Surgisis (porcine intestinal submucosa) and Gore-tex when used for Congenital DH.^28^ Other small studies have suggested that biological meshes may yet have a role. One study of 46 patients indicated that primary closure with biologic mesh reinforcement might reduce recurrence rates.^29^

Mesh involved in both HH and DH are usually placed intraperitoneally, and therefore, the potential of adhesion formation is an important consideration. Whilst the majority of studies in our review did assess adhesion formation (11/18), only 8 used any form of scoring system, with the other 3 only commenting qualitatively. An issue going forward will be identifying a singular scoring system for adhesion formation, with 4 identified in this study.

For meaningful standardisation to be imposed, there must be organisations willing to assess the evidence and develop such standards. These standards will require collaboration between surgical organisations such as the European Paediatric Surgical Association or the European Hernia society and materials testing organisations such as the ASTM. Such regulations are not beyond the scope of such organisations; the ASTM having already developed a host of regulations for medical device testing,^30^ even including regulations for testing guinea pigs for contact allergens.^31^ Some organisations already appear to be heading toward standardisation as discussed by Liu et al.^32^ who describe the development of potential new regulations by the Chinese NMPA.

Whilst the minutiae of medical device testing may be outside the “wheelhouse” of many surgeons, both clinicians and industry stand to benefit from a broader understanding of the subject. Medical devices are often advertised or sold directly to clinicians at hospital sites and conferences. Such points of sale represent an opportunity for clinicians to scrutinise evidence presented by medical device companies. We hope the findings of this review may empower its readership to enquire more deeply when discussing the sale of mesh products.

Whilst pre-clinical testing will benefit from standardisation, ongoing post-market surveillance remains a crucial tool to ensure such testing yields improved outcomes. Such surveillance has been championed by organisations such as the Danish Hernia Registry, as other nations develop their own hernia registries. It is not yet clear if such registries will be developed for HH and DH devices.

### Limitations

The strength of our results is limited by the low number of studies within the review (only 18). Our review was also limited to English language studies from the last 20 years. This limited time frame was chosen intentionally to identify the most recent patterns within the field.

## Conclusion

Over the last 20 years, only 18 peer-reviewed studies have been published testing hernia mesh on in vivo HH or DH models. Most studies appear to use similar comparators in terms of histology and mechanical testing, but how they measure these remains varied. Various scoring systems exist for histological outcomes as well as adhesion scoring. Further efforts could be made at this stage to standardise the field and therefore improve further data sets.

## Supplementary Information

Below is the link to the electronic supplementary material.

Supplementary file1 (DOCX 16 KB)

Supplementary file2 (DOCX 31 KB)

## Data Availability

Given that this article is a review, there is no requirement for a data availability statement.
