# Supplementary material for: A Systematic Review Examining the Experimental Methodology Behind In Vivo Testing of Hiatus Hernia and Diaphragmatic Hernia Mesh
Source: J Gastrointest Surg. 2021 Dec 21;26(3):684–92. doi: 10.1007/s11605-021-05227-3 (PMC8927034; doi:10.1007/s11605-021-05227-3)
Supplement: Supplementary file 1 — Supplementary file1 (DOCX 16 KB) [file 11605_2021_5227_MOESM1_ESM.docx]

***Supplementary figure 1: Search strategies for Embase and MEDLINE:***

***Embase:***

| 1. Hiatus hernia/ or diaphragmatic hernia/ or diaphragm hernia/ |  |
| --- | --- |
| 2. ((diaphragmatic or hiatus or paraoesophageal or paraesophageal or diaphragm) adj3 hernia*).tw. |  |
| 3. 1 or 2 |  |
| 4. surgical mesh/ or mesh plug/ or nonabsorbable mesh/ or titanium mesh/ or transabdominal mesh/ |  |
| 5. mesh*.tw. |  |
| 6. 4 or 5 |  |
| 7. materials testing/ |  |
| 8. test*.tw. |  |
| 9. assess*.tw. |  |
| 10. compar*.tw. |  |
| 11. measur*.tw. |  |
| 12. 7 or 8 or 9 or 10 or 11 |  |
| 13. 3 and 6 and 12 |  |
| 14. limit 13 to (english language and yr="2000 - 2020") |  |
| 15. limit 14 to animal studies |  |

***MEDLINE:***

| \| 1. hernia, diaphragmatic/ or hernias, diaphragmatic, congenital/ or hernia, diaphragmatic, traumatic/ or hernia, hiatal/ \|  \| \| --- \| --- \| \| 2. ((hiatal or hiatus or diaphragmatic or paraesophageal or paraoesophageal or diaphragm) adj3 hernia*).tw. \|  \| \| 3. 1 or 2 \|  \| \| 4. surgical mesh/ or mesh plug/ or nonabsorbable mesh/ or titanium mesh/ or transabdominal mesh/ \|  \| \| 5. mesh*.tw. \|  \| \| 6. 4 or 5 \|  \| \| 7. Materials Testing/ \|  \| \| 8. test*.tw. \|  \| \| 9. compar*.tw. \|  \| \| 10. measur*.tw. \|  \| \| 11. assess*.tw. \|  \| \| 12. 7 or 8 or 9 or 10 or 11 \|  \| \| 13. 3 and 6 and 12 \|  \| \| 14. limit 13 to animals \|  \| \| 15. limit 14 to (english language and yr="2000 - 2020") \|  \| |  |
| --- | --- | --- | --- | --- | --- | --- | --- | --- | --- | --- | --- | --- | --- | --- | --- | --- | --- | --- | --- | --- | --- | --- | --- | --- | --- | --- | --- | --- | --- | --- | --- |
|  |  |
|  |  |
|  |  |
|  |  |
|  |  |
|  |  |
|  |  |
|  |  |
|  |  |
|  |  |
|  |  |
|  |  |
|  |  |
|  |  |
